# RelTime Rates Collapse to a Strict Clock When Estimating the Timeline of Animal Diversification

**DOI:** 10.1093/gbe/evx079

**Published:** 2017-05-01

**Authors:** Jesus Lozano-Fernandez, Mario dos Reis, Philip C.J. Donoghue, Davide Pisani

**Affiliations:** 1 School of Earth Sciences, Life Sciences Building, University of Bristol, United Kingdom; 2 School of Biological Sciences, Life Sciences Building, University of Bristol, United Kingdom; 3 School of Biological and Chemical Sciences, Queen Mary University of London, United Kingdom

**Keywords:** molecular clocks, Bayesian relaxed-clock methods, RelTime, animal evolution, fossil calibrations

## Abstract

Establishing an accurate timescale for the history of life is crucial to understand evolutionary processes. For this purpose, relaxed molecular clock models implemented in a Bayesian MCMC framework are generally used. However, these methods are time consuming. RelTime, a non-Bayesian method implementing a fast, ad hoc, algorithm for relative dating, was developed to overcome the computational inefficiencies of Bayesian software. RelTime was recently used to investigate the timing of origin of animals, yielding results consistent with early strict clock studies from the 1980s and 1990s, estimating metazoans to have a Mesoproterozoic origin—over a billion years ago. RelTime results are unexpected and disagree with the largest majority of modern, relaxed, Bayesian molecular clock analyses, which suggest animals originated in the Tonian-Cryogenian (less that 850 million years ago). Here, we demonstrate that RelTime-inferred divergence times for the origin of animals are spurious, a consequence of the inability of RelTime to relax the clock along the internal branches of the animal phylogeny. RelTime-inferred divergence times are comparable to strict-clock estimates because they are essentially inferred under a strict clock. Our results warn us of the danger of using ad hoc algorithms making implicit assumptions about rate changes along a tree. Our study roundly rejects a Mesoproterozoic origin of animals; metazoans emerged in the Tonian-Cryogenian, and diversified in the Ediacaran, in the immediate prelude to the routine fossilization of animals in the Cambrian associated with the emergence of readily preserved skeletons.

## Introduction

Timescales are essential to evolutionary biology, calibrating biological processes to human and geological timescales and elucidating the tempo and mode of evolution, from viral strains (Worobey et al. 2016) to the entire Tree of Life (Shih and Matzke 2013). However, the best approach for deriving accurate evolutionary timescales remains unclear. Molecular clock methods have developed dramatically from early approaches that assumed a “strict” clock of unvarying rate that yielded divergence time estimates for animals that were often double the age of the oldest fossil evidence (Benton and Ayala 2003). There is now a diversity of increasingly complex Bayesian “relaxed” clock models that do not assume a constant substitution rate, and integrate not just fossil age-uncertainty (Rannala and Yang 2007; Donoghue and Yang 2016; dos Reis et al. 2016) but also fossil phylogenetic-uncertainty (Ronquist, Klopfstein, et al. 2012; Heath et al. 2014). These new Bayesian methods generally find divergence times for animals that are in much closer accord with the fossil record (Erwin et al. 2011; dos Reis et al. 2016) than those obtained under the early, strict-clock-based methods (Runnegar 1982; Wray et al. 1996). While the diversification of methods is welcome, their increasing complexity and computational cost make their application to genome-scale datasets difficult. RelTime (Tamura et al. 2012), a non-Bayesian method implementing a fast but ad hoc algorithm to assign rates to the branches of a phylogenetic tree, has been developed specifically to overcome the computational inefficiency of contemporary Bayesian molecular clock methods. Because RelTime estimates rates of evolution quickly, it can process genome scale datasets for hundreds of taxa a thousand times faster than the most efficient Bayesian molecular clock method (Tamura et al. 2012).

RelTime eschews fossil calibrations, estimating relative, rather than absolute, evolutionary timescales. RelTime relative timescales can be transformed into absolute timescales using calibration anchors that are considered reliable a priori, to re-scale branch lengths to absolute time (Tamura et al. 2012). Advocates of RelTime claim that by eschewing fossils while estimating rates, RelTime avoids the negative impact of “flawed” calibrations in divergence time estimation (Tamura et al. 2012; Battistuzzi et al. 2015; Kumar and Hedges 2016). In a series of recent studies, RelTime has been benchmarked against Bayesian divergence time analyses, recovering comparable results in a fraction of the time (Mello et al. 2017). However, in reproducing analyses of the timing of the animal diversification using the dataset of Erwin et al. (2011), Battistuzzi et al. (2015) recovered a much older Mesoproterozoic estimate for the origin of animals, akin to the results from early studies that relied on strict clock methods (Runnegar 1982; Wray et al. 1996). Battistuzzi et al. (2015) estimated that animals diverged more than half a billion years before the first animal fossils, and hundreds of millions of years earlier than all contemporary Bayesian divergence time analyses (Parfrey et al. 2011; dos Reis et al. 2015; Sharpe et al. 2015) which, in agreement with Erwin et al. (2011), suggest that animals emerged in the Neoproterozoic. Battistuzzi et al. (2015) attributed the difference between their results and those of Erwin et al. (2011) to the use of “flawed calibrations” in the study of Erwin et al. (2011). This is surprising given the congruence between the results of Erwin et al. (2011) with Sharpe et al. (2015), dos Reis et al. (2016) and Parfrey et al. (2011), which used different sets of calibrations and different root priors. Here we show that the disparity between the results of Erwin et al. (2011) and Battistuzzi et al. (2015) was not caused by the use of “flawed” calibrations but, rather, by the fact that RelTime cannot adequately relax the clock, along the internal branches of the animal phylogeny. The failure of RelTime to relax the clock for Erwin et al. (2011) dataset indicates that while this software is undoubtedly fast, it is not always reliable when establishing evolutionary timescales in deep time. As such, we advocate the use of computationally slower, but more accurate, Bayesian methods like those used by Erwin et al. (2011) and dos Reis et al. (2015). These methods can better relax the clock, and largely agree that animals emerged in the Tonian-Cryogenian and diversified in the Ediacaran, in the immediate prelude to the routine fossilization of animals in the Cambrian that is associated with the emergence of readily preserved skeletons.

## Results and Discussion

### RelTime and Phylobayes Relative Divergence Times Differ Significantly

**Fig. 1.**
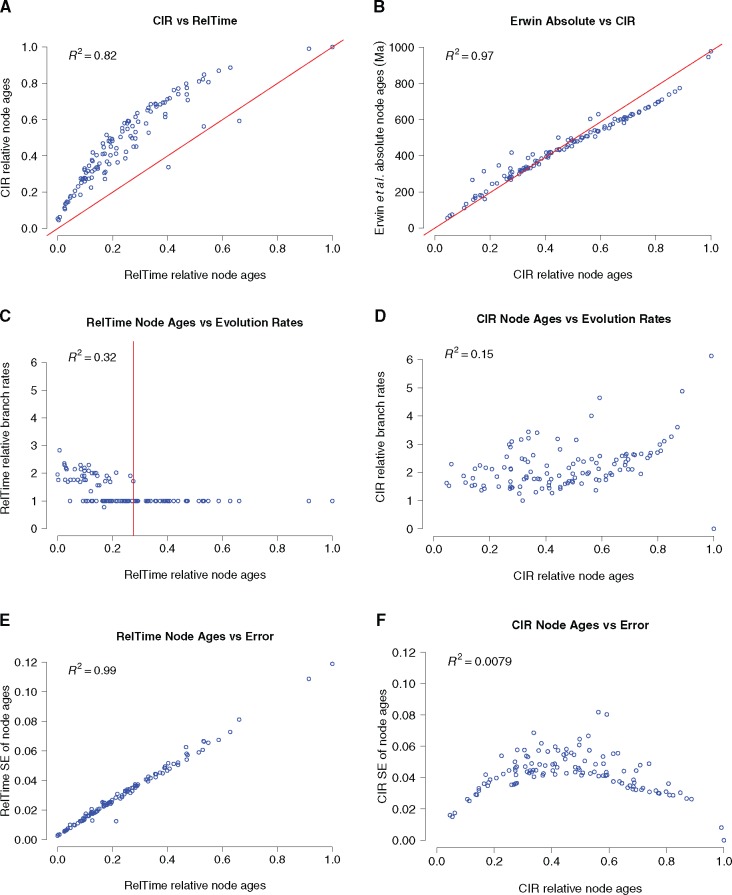
—(*a*) RelTime-inferred relative divergence times versus Bayesian relative divergence times estimated in this study under the autocorrelated, relaxed, CIR clock model. (*b*) Relative divergence times estimated in this study under the autocorrelated, relaxed, CIR clock model versus absolute divergence times re-estimated under the 24 fossil calibrations and root age prior of (Erwin et al. 2011). The red line connects the maximum and minimum values on the scatter plot. (*c*) Relative node ages versus the corresponding relative branch rates estimated using RelTime. The red line indicates the relative depth in the animal phylogeny after which all Reltime-inferred relative rates are assigned the same, constant rate. The rates assigned to branches deeper than the red line in Panel C are equal to one, which is the median rate for the dataset. (*d*) Relative node ages versus corresponding relative branch rates estimated using Bayesian inference under the autocorrelated, relaxed, CIR clock model. Under the CIR model branch rates vary along the entire tree. (*e*) Node ages versus SE for RelTime-inferred relative rates of evolution. (*f*) Relative nodes ages versus SE for Bayesian relative divergence times inferred using the CIR clock. SE, standard error. Scatter plots have been generated in R. In all panels (*a–f*), the values of *R*^2^ (the square of the linear correlation coefficient) are given. RelTime values have been normalised to one.

Regression analyses indicate that RelTime inferred relative divergence times are clearly not proportional to the corresponding Bayesian relative divergence times (fig. 1*a*). Crucially, there is no difference in the way in which RelTime-inferred relative divergence times disagree with our Bayesian relative age estimates, and with the absolute divergence times estimated by Erwin et al. (2011) (contrast fig. 1*a* here with fig. 1 in Battistuzzi et al. 2015). Conversely, our Bayesian relative divergence times are approximately proportional to the absolute divergence times of Erwin et al. (2011) (fig. 1*b*). This unambiguously demonstrates that the discrepancy between the results of Battistuzzi et al. (2015) and Erwin et al. (2011) cannot have been caused by the calibrations used by Erwin et al. (2011). Instead, the disparity in relative clade ages must be a consequence of the fundamentally different way in which RelTime and Phylobayes calculate rates of evolution.

### RelTime and Phylobayes Relative Rates Dates—Which Are the More Reliable?

**Fig. 2.**
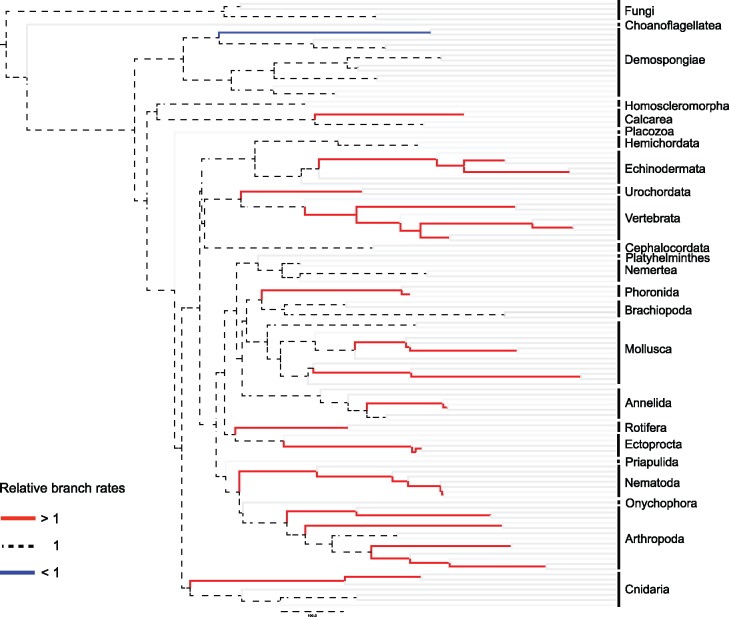
—A graphical representation of how RelTime-inferred relative rates change along the phylogeny. This figure illustrates that all rate changes inferred by RelTime for nonterminal branches but one are rate accelerations that happened towards the tips of the tree. Reltime effectively assumes clock-like evolution across the deep branches of the Opisthokonta.

The fundamental difference between relative divergence times estimated using Phylobayes and RelTime begs the question of which set of relative rates and dates is the more reliable. We investigated how inferred rates of evolution change as the tree is traversed from the tips to the root. We found that RelTime estimates of relative rates do not vary along the entire tree (fig. 1*c*), differently from Bayesian estimates of relative rates (fig. 1*d*). The distribution of RelTime relative rates across the tree is highly asymmetrical, with rate changes concentrated towards the tips (e.g. within Mollusca, Vertebrata, Bryozoa, Nematoda, Arthropoda, Cnidaria, Echinodermata). Rootward, RelTime relative rates settle to a value of 1 (the median value for the dataset) and are no longer relaxed (fig. 1*c*). That is, as we move towards the root of the tree, RelTime stops inferring rate changes and instead infers a clock-like evolutionary rate (figs. 1*c* and 2). It is also surprising that all RelTime inferred rate changes (bar one) are rate increments (fig. 2); only one rate decrease is inferred, within the silicean sponges, a clade that is otherwise assumed to have evolved under a strict clock (fig. 2). Battistuzzi et al. (2015) inferred the majority of their RelTime relative dates under a strict molecular clock (see also Lozano-Fernandez et al. 2016), implying that opisthokont evolution was mostly clock-like, with the few deviations from this pattern representing, in all but one case, tipward rate accelerations.

### Errors Around RelTime Relative Rate Estimates Increase as the Tree is Traversed from the Tips to the Root

The distribution of error associated with RelTime and Phylobayes relative rate estimates appears informative in attempting to understand the discrepancy between the results of Erwin et al. (2011) and Battistuzzi et al. (2015). Standard Errors (SEs) around RelTime relative rates increase linearly with the age of the node in the tree (*R*^2^=0.99), with the largest SE associated with the root (fig. 1*e*). While this is not unexpected, a different pattern is associated with Phylobayes relative rates (fig. 1*f*), where errors do not increase linearly with node age. According to Tamura et al. (2012), RelTime uses estimated SEs around the relative rates of parent and daughter branches to determine whether they should be allocated the same or a different relative rate. While it was not the aim of this study to investigate why RelTime infers clock-like rates deep in animal history (figs. 1*c* and 2), we speculate that the increasing error around relative rate estimates associated with branches closest to the root (fig. 1*e*) might imply that RelTime is biased in the way it relaxes the clock. That is, the closer branches are to the root, the lower the likelihood that RelTime will identify them as having different rates. This would explain the distribution of RelTime relative rates in figures 1*c* and 2, and why Battistuzzi et al. (2015) estimated ages for the deepest part of the opisthokont (and metazoan) history that are congruent with the obsolete strict clock estimates of Runnegar (1982) and Wray et al. (1996).

### Metazoans Have a Neoproterozoic Origin

**Fig. 3.**
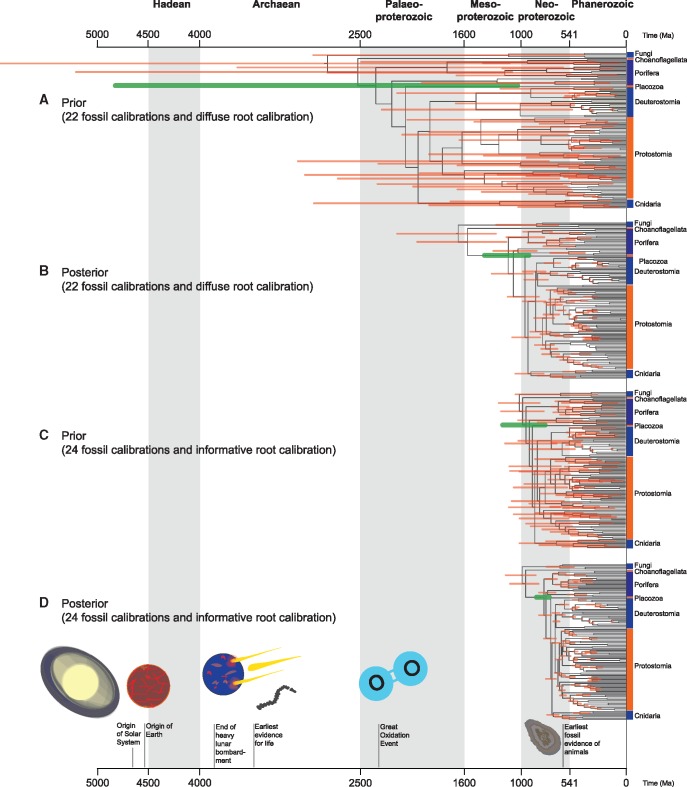
—Time trees showing marginal priors and posterior divergence time estimated for the metazoan tree of life under the CIR clock model. (*a*) Marginal priors of divergence times using the 22 fossil-calibration set (table 1). (*b*) Posterior divergence times using the 22 fossil-calibrations set (table 1). (*c*) Marginal priors of divergence times using the 24 fossil-calibration set (table 1). (*d*) Posterior divergence times estimates using the 24 fossil-calibration set (table 1). In (*a*) and (*b*), the calibration density on the root age is exponential with mean = 1,000 Myr. In (*c*) and (*d*), the calibration density on the root age is gamma with mean = 1,000 Ma and SD = 100 Ma. Nodes are drawn at the posterior means and horizontal thick bars represent the 95% highest posterior density (HPD) intervals. The HPD interval bar for the crown-metazoan node age is highlighted in green. Images on the bottom x axis depicts relevant geological and biological events. It is noticeable that irrespective of the fossil calibrations and node age prior used, posterior divergence times always tend to sit at the right end (i.e. young ages) of the prior distribution. The results presented here represent strong evidence rejecting the Battistuzzi et al. (2015) hypothesis that animals had a long cryptic history that went unrecorded in the fossil record.

The marginal node age priors from Battistuzzi et al. (2015) demonstrate that their Bayesian analyses could not meaningfully discriminate between a Mesoproterozoic or a Neoproterozoic origin of animals (see tables 1 and 2 and fig. 3). This occurs for two different but related reasons. First, the exponential density used by Battistuzzi et al. (2015) to calibrate the root age is unreasonable. This exponential density is very diffuse, with a mean of 1,000 Ma and a 95% inter-quantile range of 3,687–26 Myr. These numbers might seem conservative, as they might suggest that the authors assumed absolute ignorance about the time of origin of Opisthokonta—Erwin et al. (2011) used Fungi as outgroups. However, this view is misleading since the specified root prior is transformed in the construction of the joint time prior for the tree to accommodate the fact that ancestral nodes must be older than their descendants (Inoue et al. 2010; Warnock et al. 2012). In the specific case of Battistuzzi et al. (2015), truncation caused the marginal prior for the root age to be skewed towards the older range of ages, resulting in a prior mean of 2,810 Ma (not 1,000 Ma) and a 95% prior interval of 6,046–1,091 Myr (not 3,687–26 Ma). Accordingly, the Battistuzzi et al. (2015) effective root prior assigns nonnegligible prior probabilities of the last common opisthokont ancestor being older than the Solar System. Similarly, other node ages also present effective priors that extend unreasonably back in time: The 95% prior age interval for crown Metazoa is 4,611–1,091 Myr, 4,102–1,015 Myr for crown Eumetazoa, and 3,724–898 Myr for crown Bilateria . All of these node age priors encompass times that exceed the oldest direct evidence of Life on Earth (∼3,700 Ma; Nutman et al. 2016). At the same time, the effective priors of Battistuzzi et al. (2015) assigned a vanishingly small cumulative probability (prior probability < 0.025) to a Neoproterozoic last common ancestor of Metazoa, imposing a very strong bias in favour of a pre-Neoproterozoic origin of animals in their Bayesian analyses. In other words, in attempting to use a seemingly uninformative calibration on the root age, Battistuzzi et al. (2015) effectively assigned a highly and inappropriately informative prior. In contrast, the marginal priors associated with the analyses of Erwin et al. (2011) (see tables 1 and 2, and fig. 3) assigned comparable prior probabilities to both a Mesoproterozoic and a Neoproterozoic last common animal ancestor.

**Table 1:** Summary of the Bayesian Analyses Carried Out Using Two Sets of Fossil Calibrations

Calibration Set	Substitution Model	Clock Model	Number of Calibrations	Root age Calibration	Soft Bound
Erwin	CAT-GTR+G	CIR	24	Gamma with mean=1,000 Ma and SD = 100 Myr	5%
Battistuzzi	CAT-GTR+G	CIR	22	Exponential* with mean=1,000 Ma	5%

Note.—Legend: 24 = The original 24 calibration set of Erwin et al. (2011). These calibrations are available, with their palaeontological justifications, in table S4 of the original study of Erwin et al. (2011). 22** **= The calibration set used by Battistuzzi et al. (2015) and composed of all the calibrations of Erwin et al. (2011) with the exclusion of two that Battistuzzi et al. (2015) deemed to be flawed following their RelTime analysis. Excluded calibrations are: (1) The soft maximum on the crown Demospongiae, set by Erwin et al. (2011) to 713 Ma based on molecular biomarker evidence (Love et al. 2009; Sperling et al. 2010; Love and Summons 2015). (2) The soft maximum on the origin of Ambulacraria, set to 565 Ma based on arguments presented in Peterson et al. (2008). Note that this soft maximum has recently been reevaluated by Benton et al. (2015), but for the scope of our study this is not relevant as the absolute divergence time estimated by Erwin et al. (2011) for this clade is fully compatible with the Benton et al. (2015) constraint. *Note: Battistuzzi et al. (2015) described their root calibration as a gamma density with mean = 1,000 Ma and SD = 1,000 Myr. This gamma density has shape parameter = 1, and it is thus an exponential distribution of mean = 1,000 Ma.

**Table 2:** Divergence Times for Key Nodes as Estimated Under the 22-Fossil Calibration Set and Root Prior of Battistuzzi et al. (2015) and the 24-Fossil Calibration Set and Root Prior of Erwin et al. (2011) (see table 1 for details)

Calibration Set	MCMC Run	Root Age (Ma)	Crown-Metazoa (Ma)	Crown-Eumetazoa (Ma)	Crown-Bilateria (Ma)
Battistuzzi	Marginal priors	2,810 (6,046–1,091)	2,354 (4,611–1,045)	2,075 (4,102–1015)	1,846 (3,724–898)
Battistuzzi	Posterior	1,604 (2,096–1171)	1,126 (1,349–922)	964 (1,126–825)	862 (988–757)
Erwin	Marginal priors	1,026 (1,235–841)	960 (1,178–732)	875 (1,130–629)	852 (1,118–615)
Erwin	Posterior	986 (1,134–858)	778 (853–721)	701 (765–659)	672 (716–637)

Note.—Legend: **Battistuzzi **=** **Mean node ages (and 95% HPDs) inferred using the reduced set of 22 fossil calibrations and the diffuse root age calibration of Battistuzzi et al. (2015) (see “Materials and Methods” section and table 1). **Erwin **=** **Mean node ages (and 95% HPDs) inferred using the same set of 24 fossil calibrations and root age calibration used in the in the original Bayesian analysis of Erwin et al. (2011) (see “Materials and Methods” section and table 1). **Marginal Priors** are calculated by running the MCMC chain with no data according to the calibration set used (table 1). **Posteriors** are calculated by running the MCMC chain with the molecular data and the calibration set used (table 1). Whereas the prior mean age of the crown Metazoa in Battistuzzi et al. (2015) study was deep in the Neoarchaean 2,354 Ma before the Neoproterozoic-Mesoproterozoic boundary, the mean prior crown age for the same node in the study of Erwin et al. (2011) is 960 Ma at all effect centered on the Neoproterozoic-Mesoproterozoic boundary. It follows that while the calibrations and root prior of Erwin et al. (2011) allowed a fair test of whether the age of animals happened in the Mesoproterozoic or in the Neoproterozoic, those of Battistuzzi et al. (2015) did not, biasing the results towards a Mesoproterozoic origin of animals.

Absolute divergence time analyses performed under the priors used by Battistuzzi et al. (2015), estimated the origin of crown Metazoa at 1,349–922 Myr. The prior probability for these ages is strongly skewed towards the younger end of the effective prior distribution used by Battistuzzi et al. (2015) (fig. 3), indicating that the data are informative. Crucially, despite the effective priors imposing a strong bias towards the inference of a pre-Neoproterozoic last animal common ancestor (fig. 3), analyses performed under Battistuzzi et al. (2015) priors failed to reject a Neoproterozoic (1,000–541 Ma) origin of animals. Using the specified priors from Battistuzzi et al. (2015), neither a Mesoproterozoic nor a Neoproterozoic origin of animals can be rejected.

The effective priors of Battistuzzi et al. (2015) are inadequate to discriminate between alternative hypotheses for the origin of animals. This is not surprising as the approach employed by Battistuzzi et al. (2015) to identify “flawed” calibrations, is itself flawed. Their approach assumes that if absolute divergence times and RelTime inferred relative divergence times disagree, it must be because the absolute divergence times were misled by “flawed” fossil calibrations. That is, the Battistuzzi et al. (2015) approach implicitly assumes the infallibility, not of the genomic record, but of the RelTime interpretation of this record. However, discrepancies between relative and absolute divergence time estimates should be anticipated when, as here, the clock is violated and a molecular clock method that does not relax the clock is used. When the clock is violated, calibrations provide local checks on rate variation, which are crucial to estimate accurate rates and thus accurate divergence times in relaxed molecular clock analyses (Hugall et al. 2007; and Warnock et al. 2015). As such, the exclusion of calibrations, justified on explicit phylogenetic, stratigraphic and palaeontological evidence, in soft bounded, relaxed, molecular clock analyses is unjustified and potentially deleterious.

Battistuzzi, Kumar, Hedges and colleagues (Tamura et al. 2012; Battistuzzi et al. 2015; Kumar and Hedges 2016) suggested that the ability of RelTime to estimate relative divergence times is a special feature of their software. However, all standard Bayesian relaxed molecular clock software can be used to estimate both relative and absolute divergence times and rates [e.g. MCMCTree (Yang 2007), Phylobayes (Lartillot et al. 2009), BEAST (Drummond and Rambaut 2007) and MrBayes (Ronquist, Teslenko, et al. 2012)]. We do not deny the potential utility of relative divergence times (see Loader et al. 2007), but we question the reliability of RelTime, since our study clearly demonstrates that, at the least for the dataset chosen by Battistuzzi et al. (2015), the RelTime method have proven highly inaccurate. A second important feature of RelTime, according to Tamura et al. (2012), is that it eliminates the requirement to specify a statistical distribution of rates, in contrast to Bayesian methods where an explicit distribution such as the independent log-normal or the CIR process is required. However, this does not make RelTime assumption-free. To calculate rate changes along the branches of a tree, the RelTime algorithm uses specific mathematical formulae representing strong statements about how rate variation can occur, even if the formulation is not based on explicit model assumptions. The result is that implicit assumptions are being made and, we contend, implicit assumptions are more problematic than explicit assumptions, as their implications are not clear to either the algorithm designer or the end user. Here we have shown that RelTime inadvertently infers clock-like evolution on the ancestral branches of animal and opisthokont phylogeny, which defeats the whole purpose of designing a method to account for rate variation in the first place.

## Conclusions

The conclusions drawn by Battistuzzi et al. (2015) rely on results derived 1) using the RelTime method for divergence time estimation which failed to relax the clock in modeling early animal evolution, 2) a flawed strategy to eliminate incongruous fossil calibrations, and 3) the imposition of an arbitrary, highly (but spuriously) informative root prior that favoured the recovery of a Mesoproterozoic animal ancestor. Irrespective of all the above, their key conclusion, that animals have a Mesoproterozoic origin, is invalid since it does not encompass the uncertainty associated with their own analysis, which yielded a Mesoproterozoic–Neoproterozoic estimate for the origin of animals.

The RelTime results obtained by Battistuzzi et al. (2015) are a blast from the past, deriving from the failure of RelTime to relax the clock in deep time—mirroring the flaws of the earliest strict molecular clock methods and analyses which have already been roundly rejected (Graur and Martin 2004). The precise timing of early animal evolution remains obscured by uncertainty associated with concomitantly imprecise fossil calibrations (Cunningham et al. 2017). However, all recent Bayesian analyses (dos Reis et al. 2015; Peterson et al. 2008; Sperling et al. 2010; Erwin et al. 2011; Parfrey et al. 2011; Sharpe et al. 2015) have the statistical power to reject a long, cryptic, Mesoproterozoic history, instead converging on an albeit loosely constrained Neoproterozoic origin of animals.

## Materials and Methods

We used a Bayesian relaxed-clock method to estimate relative rates of evolution and relative divergence times for Erwin et al. (2011) dataset, and compared these estimates against relative rates and ages estimated with RelTime for the same dataset by Battistuzzi et al. (2015). In addition we re-estimated absolute Bayesian divergence times using the same fossil calibrations as in Erwin et al. (2011), excluding the calibrations deemed to be “flawed” by Battistuzzi et al. (2015), a soft maximum of the crown Desmospongiae (set by Erwin et al. (2011) at 713 Ma—table 1 for details) and a soft maximum on the origin of Ambulacraria (set by Erwin et al. (2011) to at 565 Ma—table 1 for details), based on the absence of a linear relationship between the Bayesian absolute divergence times and the relatives ages produced by RelTime. Phylobayes version 4.1 (Lartillot et al. 2009) was used for all Bayesian molecular clock analysis. The autocorrelated, relaxed, CIR clock model (Lepage et al. 2007) was used to maintain consistency across compared studies, as this model was also used by Erwin et al. (2011) and in all of the Bayesian analyses of Battistuzzi et al. (2015). To obtain Bayesian relative divergence times, we simply fixed the root of the tree used by Erwin et al. (2011) to have an age of one and omitted all other calibrations. We reason that if Battistuzzi et al. (2015) are correct (that the absolute divergence times estimated by Erwin et al. (2011) are biased by the use of flawed fossil calibrations), then relative ages estimated using the Bayesian method should be proportional to relative ages estimated using RelTime. This should not be the case for the absolute Bayesian divergence times estimated using the same calibrations of Erwin et al. (2011).

### Comparing Bayesian and RelTime Relative Ages and Rates

RelTime ages and rates from Battistuzzi et al. (2015) were kindly provided by Fabia Battistuzzi. Bayesian relative ages were compared with those inferred by RelTime (Battistuzzi et al. 2015) using standard regression analyses. Bayesian relative ages were also compared with the absolute ages we obtained in our reanalyses of the Erwin et al. (2011) dataset. Subsequently, we explored how Bayesian and RelTime relative rates and their attendant errors change as the tree is traversed from the tips to the root.

### Testing the Validity of the Absolute Divergence Times of Battistuzzi

Battistuzzi et al. (2015) asserted that the root age calibration density used by Erwin et al. (2011), a gamma distributed prior with a mean of 1,000 Ma and SD of 100 Myr, “unduly restricted the root constraint” and biased the corresponding node age estimates towards the present. Accordingly, to validate their RelTime-estimated timescale of animal evolution, Battistuzzi et al. (2015) estimated new, absolute, Bayesian divergence times using an exponential density with a mean of 1,000 Ma for the datasets of Erwin et al. (2011), implicitly assuming that this new prior would not unduly restrict the root age. However, Battistuzzi et al. (2015) did not present evidence to support their implicit assumption. In addition, for their Bayesian re-analyses, Battistuzzi et al. (2015) used only 22 of the 24 fossil calibrations of Erwin et al. (2011), as the two remaining calibrations from Erwin et al. (2011) were identified as “flawed” using their RelTime-based approach to validate fossil calibrations. Here we compared Bayesian estimates of node ages under the gamma distributed root age prior of Erwin et al. (2011) with 24 fossil calibrations, against the exponential root age prior of (Battistuzzi et al. 2015) with 22 fossil calibrations, and assessed whether these two analyses were adequate to discriminate between a Mesoproterozoic and a Neoproterozoic origin of animals. We first visualised the marginal priors on the root and node ages generated under both sets of calibrations by running the MCMC Bayesian analyses without sequence data. Subsequently, we re-estimated divergence times under each set of calibrations and root age prior and compared the results to each other, as well as against the respective marginal priors. Tracecomp was used to determine if the MCMC Bayesian molecular clock analyses were run to an acceptable level of convergence (see Phylobayes manual).

The key priors and parameters from each of the two absolute divergence time analyses (including details of the fossil calibrations) are summarised in table 1.
